# Ex vivo model predicted in vivo efficacy of CFTR modulator therapy in a child with rare genotype

**DOI:** 10.1002/mgg3.1656

**Published:** 2021-03-13

**Authors:** Vito Terlizzi, Felice Amato, Chiara Castellani, Beatrice Ferrari, Luis J. V. Galietta, Giuseppe Castaldo, Giovanni Taccetti

**Affiliations:** ^1^ Cystic Fibrosis Regional Reference Center, Department of Paediatric Medicine Anna Meyer Children's University Florence Italy; ^2^ Department of Molecular Medicine and Medical Biotechnology University of Naples Federico II; ^3^ CEINGE – Advanced Biotechnologies Naples Italy; ^4^ Telethon Institute of Genetics and Medicine (TIGEM Pozzuoli Italy); ^5^ Department of Translational Medical Sciences University of Naples Federico II Napoli Italy

**Keywords:** children, cystic fibrosis, lumacaftor/ivacaftor, nasal brushing

## Abstract

**Background:**

New drugs that target the basic defect in cystic fibrosis (CF) patients may now be used in a large number of patients carrying responsive mutations. Nevertheless, further research is needed to extend the benefit of these treatments to patients with rare mutations that are still uncharacterized in vitro and that are not included in clinical trials. For this purpose, ex vivo models are necessary to preliminary assessing the effect of CFTR modulators in these cases.

**Method:**

We report the clinical effectiveness of lumacaftor/ivacaftor therapy prescribed to a CF child with a rare genetic profile (p.Phe508del/p.Gly970Asp) after testing the drug on nasal epithelial cells. We observed a significant drop of the sweat chloride value, as of the lung clearance index. A longer follow‐up period is needed to define the effects of therapy on pancreatic status, although an increase of the fecal elastase values was found.

**Conclusion:**

Drug response obtained on nasal epithelial cells correlates with changes in vivo therapeutic endpoints and can be a predictor of clinical efficacy of novel drugs especially in pediatric patients.

## Graphical abstract

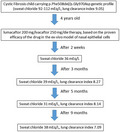

## 1 INTRODUCTION

Cystic fibrosis (CF, MIM: 219700) is an autosomal recessive genetic disease characterized by mutations in the *cystic fibrosis transmembrane conductance regulator* (*CFTR*, MIM: 602421) gene. To date, 352 *CFTR* mutations are known to be CF‐causing (https://cftr2.org/). They are classified according to the impact they have on the synthesis, processing, or function of the CFTR protein. New drugs that target the basic defect in CF have provided hope for patients and progress in the development of such drugs has been substantial over the past decade. The first class of drugs successfully developed have been CFTR potentiators such as ivacaftor (IVA), which augment channel gating to restore 30–50% of CFTR‐mediated anion transport in patients with mutations that impair gating and residual function of the protein (Davies et al., (2013); Salvatore et al., 2020). At present, IVA (Kalydeco^®^) is approved in the United States for children aged 4 months or older who have at least one responsive mutation in *CFTR*, based on clinical and/or in vitro assay data. Kalydeco is also approved in the European Union for the treatment of CF patients aged 6 months and older with gating mutations.

The CFTR correctors correct the processing and trafficking defect of the p.Phe508del—CFTR protein to enable it to reach the cell surface. The combination of a single corrector, either lumacaftor (LUM) or tezacaftor, with the potentiator IVA improves clinical outcomes, including lung function and the rate of pulmonary exacerbations (Taylor‐Cousar et al., (2017)).

The newest CFTR modulator is elexacaftor/tezacaftor/IVA (Trikafta; Middleton et al., 2019) It was recently approved by the Food and Drug Administration and by the European Medicine Agency for the treatment of CF in patients 12 years of age or older homozygous, or compound heterozygous for the Phe508del and a minimal‐function CFTR mutation. This triple combination helps the CFTR protein perform better than other modulators and may be used in a greater number of patients with CF (Middleton et al., 2019). Nevertheless, the prevalence of p.Phe508del varies in different countries and only 45% of patients followed at CF center of Florence, Italy, carry at least one copy of such mutation, while 18% have a rare mutation (Taccetti et al., 2020; Terlizzi et al., 2018, 2019). For this reason, further research to extend the benefit of the treatment to patients with other responsive mutations is mandatory since they are usually excluded from clinical trials. Recently, *ex vivo* models like organoids (Liu et al., 2020) or nasal epithelial cells (Di Lullo et al., 2017; Mutyam et al., 2017) were developed to preliminary assessing the effect of molecular drugs in CF patients bearing rare mutations. Using nasal epithelial cells, we demonstrated that a child compound heterozygous for the p.Phe508del and for the rare p.Gly970Asp (c.2909G>A) mutation could benefit from CFTR pharmacotherapy (Amato et al., 2019). In particular, the study on nasal epithelial cells, combined with other in vitro tests to characterize the functional and molecular effects of the p.Gly970Asp mutation, suggested the LUM–IVA combination as a promising treatment. This was an important finding since patients with a similar missense mutation (p.Gly970Arg) were unresponsive to CFTR pharmacotherapy (De Boeck et al., 2014). Our ex vivo functional studies have allowed us to start the treatment and here we describe the clinical results after 9 months’ follow‐up.

## 2 CASE REPORT

The study was approved by the Ethical Committee of the University Federico II of Naples (Prof. G. Castaldo, n. 197.15) and the informed consent was obtained from parents of the child after a complete description of the aims of the study. We report the case of a 4 years and 6 months Caucasian child diagnosed with CF by positive newborn screening (blood immunoreactive trypsinogen 205 ng/ml, genotype: p.Phe508del/p.Gly970Asp, sweat chloride [SC]: 92–112 mEq/L). The weight–length ratio was at the 25th–50th percentile until the age of 2 years old. Pancreatic fecal elastase‐1 (EL‐1, normal value >200 µg/g) was assessed every 6 months showing values fluctuating in a wide range (30–280 µg/g). Pancreatic enzyme replacement therapy was started at the age of 3 years and 4 months for subsequent pathological finding of fecal EL‐1 (<100 µg/g), repeated episodes of abdominal pain and swelling with weight loss (drop of body mass index [BMI] from 15.05 to 14.71 in 3 months). After the start of this therapy, gastrointestinal symptoms disappeared but there was no increase in weight, with BMI of 14.33 on December 2019. Previous child's medical history was characterized by two pulmonary exacerbations for year needing oral antibiotics, the absence of chest computed tomography bronchiectasis in June 2019, chronic infection by methicillin‐sensitive *Staphylococcus aureus* and occasional isolation of *Stenotrophomonas maltophilia*. Furthermore, she presented an episode of acute pancreatitis characterized by abdominal pain and increased values of amylase (223 U/L) and lipase (2397 U/L) in June 2019, which resolved spontaneously. Orkambi therapy (LUM 200 mg/IVA 250 mg/die, weight 13.8 kg, BMI 14.82) began on December 2019 based on the proven efficacy of the drug in the ex vivo model of nasal epithelial cells from the patient (Amato et al., 2019). In the previous days, the child repeated SC test (Cl 114 mEq/L), fecal EL‐1 dosage (139 µg/g), and performed the lung clearance index (LCI 9.05 vs. predicted value (Amato et al., 2019) of 6.52), according to the standards of the American Thoracic Society (De Boeck et al., 2014; Fidler et al., 2020). She was evaluated after 2 weeks from the treatment: neither adverse reactions nor liver function parameters alterations or significant problems were reported. The SC dropped significantly (36 mEq/L). This figure was confirmed after 9 months of therapy in the same way with an improvement of the value of LCI and fecal EL‐1 (Table 1). No significant changes in BMI were found. Actually the child is very well, has not had any episode of respiratory exacerbation, nor CF related manifestations. She is continuing regular respiratory physiotherapy.

**TABLE 1:** Main findings found during the follow up

	Sweat chloride (mmol/L)	LCI	Relative change in LCI (%)	Faecal Elastase (µg/gr)	BMI
Before the start of the drug	114	9.05	n.a	139	14.33
After 2 weeks	36	n.a	n.a	239	14.33
After 3 months	39	8.27	−8.6	297	14.43
After 5 months	31	8.14	−10	n.a	15.25
After 9 months	38	7.09	−21.6	469	14.39

Abbreviations: BMI, body mass index; LCI: lung clearance index; n.a, not available changes between visits in LCI outcome are shown as a per cent of the value before the start of the drug (9.05).

The main findings during the follow‐up are shown in Table 1.

## 3 DISCUSSION

We report a case of successfully applying LUM/IVA therapy, started after testing the effectiveness on nasal epithelial cells sampled by nasal brushing (Amato et al., 2019) in a patient with a rare mutation, not included among those sensitive to the drug. Our major concern regarded the p.Gly970Asp mutation since previous clinical studies had shown no benefit from CFTR modulators in patients with a similar missense mutation (p.Gly970Arg; De Boeck et al., 2014). Indeed, as demonstrated by our group, and confirmed recently by Fidler et al., (2020) the c.2908G>C causes a complete absence of mature CFTR protein, due to a cryptic splicing defect. If p.Gly970Asp had also been undruggable, then the presence of a single copy of p.Phe508del would not have justified the use of available CFTR drugs. Our study showed that p.Gly970Asp is actually druggable and that LUM/IVA was an adequate combination to address the mixed trafficking/gating defects caused by the two mutations in the patient. During follow‐up, the main efficacy outcomes were the changes of the SC and the LCI. SC is one of the main diagnostic tools used in newborn screening programs and one of the main biomarkers of efficacy, in terms of improvement of CFTR protein expression/function, by modifying drugs (Robinson et al., 2018). We observed a reduction of the SC already after 2 weeks of treatment, which persisted after 9 months from the beginning of the therapy.

The LCI, measured by the multiple breath washout test, is a lung function outcome that has been shown to be more sensitive than spirometry, to correlate with airway changes seen on high‐resolution computed tomography and to detect significant treatment effects in randomized controlled trials (Accurso et al., 2014; Aurora et al., 2004; Shaw et al., 2020; Singer et al., 2013; Spruit et al., 2013). A percentage change in LCI greater than ±15% in preschool children can be considered physiologically relevant and greater than the biological variability of the test (Oude Engberink et al., 2017). In our case, we observed an improvement of LCI already after 3 months of therapy, up to a decrease of 21.6% after 9 months of therapy (Table 1).

Fecal pancreatic elastase‐1 is a useful indirect test of exocrine pancreatic function nevertheless an important intra‐patient variability in CF patients where the clinical picture is less clear was decribed (Meyts et al., 2002). Furthermore, although the pancreatic status is closely related to the *CFTR* genotype (Terlizzi et al., ,^2014^, ^2020^), pancreatic destruction can occur in most cases by 4 years of age (Gibson‐Corley et al., 2016). These data may explain the trend of elastase in our patient. CFTR modulators may decrease episodes of pancreatitis among individuals with CF with residual pancreatic function (Carrion et al., 2018) and significantly reduce pulmonary exacerbations even in patients without early lung function improvement (McColley et al., 2019). In our case, after the start of therapy, we observed an increase in elastase values and no episodes of pancreatitis neither respiratory exacerbations. Nevertheless, a longer follow‐up is necessary to confirm these data.

Given the high number of *CFTR* mutations, functional drug testing on models from patients represent a major step forward to predict effective treatments in an individual setting (Beekman, 2016). Rectal (Berkers et al., 2019) and nasal (Liu et al., 2020) organoids can act as a prospective biomarker for *in vivo* CFTR modulator responses. The model of cultured human nasal epithelial cells is a rapid, poorly invasive and effective tool to investigate the effect of novel mutations (Terlizzi et al., 2017, 2018) and to assess the effect of novel molecular therapies in each patient, especially in pediatric age. The present case demonstrates that drug response obtained on nasal epithelial cells correlates with changes *in vivo* therapeutic endpoints, such as LCI and SC values, confirming the efficacy of ex vivo testing on nasal epithelial cells as a predictor of clinical efficacy of novel drugs. Last but not least, this type of analysis allows to evaluate the functionality of the CFTR protein in the genetic background of the patient, with the advantage of evaluating its functionality even in the presence of effects deriving from modifying genes or the presence of complex alleles (Terlizzi et al., 2017).

However, these ex vivo procedures require expertise and specialized equipments that limit their widespread diffusion in the various CF clinical centers. It would be appropriate to create a network of ex vivo assay laboratories, with standardized analysis protocols, to provide fast and accurate results to CF clinical centers dealing with patients with rare mutations at the national and international levels.

## CONFLICT OF INTEREST

The authors have indicated they have no conflicts of interest relevant to this article to disclose.

## AUTHOR CONTRIBUTIONS

All authors contributed to conception and design of the paper, revised it critically, approved the final version to be published and agreed to be accountable for all aspects of the work in ensuring that questions related to the accuracy or integrity of any part of the work are appropriately investigated and resolved. Dr Vito Terlizzi and Dr Giovanni Taccetti followed the patient and wrote the manuscript; Dr Chiara Castellani and Dr Beatrice Ferrari performed the lung clearance index and interpreted the results; Prof Felice Amato and Prof Giuseppe Castaldo performed ex vivo analysis on nasal cells from the patient and were responsible for the Ethical Committee approval of the study; Prof Luis JV Galietta performed in vitro analysis. All authors approved the final manuscript as submitted and agree to be accountable for all aspects of the work.

## Data Availability

All reported data are available.
